# Applying Unbiased, Functional Criteria Allows Selection of Novel Cyclic Peptides for Effective Targeted Drug Delivery to Malignant Prostate Cancer Cells

**DOI:** 10.3390/pharmaceutics17070866

**Published:** 2025-07-01

**Authors:** Anna Cohen, Maysoon Kashkoosh, Vipin Sharma, Akash Panja, Sagi A. Shpitzer, Shay Golan, Andrii Bazylevich, Gary Gellerman, Galia Luboshits, Michael A. Firer

**Affiliations:** 1 Department of Chemical Engineering, Ariel University, Ariel 40700, Israel; annacophd@gmail.com (A.C.); maysoonk046@gmail.com (M.K.); vipins@ariel.ac.il (V.S.); galialu@ariel.ac.il (G.L.); 2 Department of Chemical Sciences, Ariel University, Ariel 40700, Israel; akash@ariel.ac.il (A.P.); andriib@ariel.ac.il (A.B.); garyg@ariel.ac.il (G.G.); 3 Department of Urology, Rabin Medical Center, Petah Tikva 49100, Israel; sagi.shpitzer@gmail.com (S.A.S.); shaygo1@gmail.com (S.G.); 4 Gray Faculty of Medical and Health Sciences, Tel Aviv University, Tel Aviv 69978, Israel; 5 Laboratory for Immunology and Cancer Biology, Adelson School of Medicine, Ariel University, Ariel 40700, Israel

**Keywords:** peptide-drug conjugates, phage display, prostate cancer, targeted drug delivery, targeted drug delivery

## Abstract

**Background:** Metastatic prostate cancer (mPrC), with a median survival of under 2 years, represents an important unmet medical need which may benefit from the development of more effective targeted drug delivery systems. Several cell surface receptors have been identified as candidates for targeted drug delivery to mPrC cells; however, these receptors were selected for their overabundance on PrC cells rather than for their suitability for targeted delivery and uptake of cytotoxic drug payloads. **Methods:** We describe a novel, unbiased strategy to isolate peptides that fulfill functional criteria required for effective intracellular drug delivery and the specific cytotoxicity of PrC cells without prior knowledge of the targeted receptor. Phage clones displaying 7-mer cyclic peptides were negatively selected in vivo and then positively biopanned through a series of parent and drug-resistant mPrC cells. Peptides from the internalized clones were then subjected to a panel of biochemical and functional tests that led to the selection of several peptide candidates. **Results:** The selected peptides do not bind PSMA. Peptide-drug conjugates (PDCs) incorporating one of the peptides selectively killed wild-type and drug-resistant PrC cell lines and patient PrC cells but not normal prostate tissue cells in vitro. The PDC also halted the growth of PC3 tumors in a xenograft model. **Conclusions:** Our study demonstrates that adding unbiased, functional criteria into drug carrier selection protocols can lead to the discovery of novel peptides with appropriate properties required for effective targeted drug delivery into target cancer cells.

## 1. Introduction

Localized prostate cancer can be treated effectively with surgery, radiotherapy, or focal therapy [1]. However, more than 10% of men diagnosed with PrC will develop metastatic disease. The combination of androgen deprivation therapy (ADT), androgen receptor pathway inhibitors, and chemotherapy, while creating a state of medical castration, has improved the overall survival of patients with metastatic hormone-sensitive prostate cancer (mHSPrC). Despite this improvement, the median survival is under 2 years [2,3,4], and the 5-year survival rate is only 30% [3,5]. Therefore, mPrC still represents an important unmet medical need.

Recent advances in the treatment of mPrC include targeted radioligand therapy, particularly directed toward the prostate-specific membrane antigen (PSMA), whose level of expression reflects disease progression and recurrence [6]. The FDA approved PSMA-labeled lutetium (^177^Lu) (Vipivotide tetraxetan) for this purpose in 2022. Nonetheless, this therapy can lead to significant side effects [7,8], possibly because PSMA is expressed at low-to-moderate levels in several normal tissues [9,10]. In addition, 5–10% of PrC cells do not express surface PSMA, and long-term ADT can lead to a significant reduction in PSMA expression [11]. PSMA, as well as other targeting ligand candidates (reviewed in Hu et al., 2024) [12], were selected because of their overabundance of PrC cells rather than for their ability to initiate effective carrier-drug uptake and intracellular drug delivery, factors that ultimately limit the efficacy of targeted drug therapy for cancer. Another factor limiting therapies targeting only one cell surface component is that PrC cells (like many other cancers) are genetically and phenotypically heterogeneous, leading to the modulation of receptor expression. Prostate cancers are often multifocal, having topographically and morphologically distinct tumor foci [13,14]. Indeed, studies have shown marked heterogeneity in the PSMA labeling of multifocal disease [15].

Several different technologies have been used to isolate cell-targeting peptides, although arguably, the most efficient of these is based on phage peptide display techniques [16]. Regarding prostate cancer, various approaches have been used to derive a pool of peptide candidates for the detection of and targeted drug delivery (TDD) to mPrC cells, but these have yet to progress to clinical evaluation [17].

Here, we describe a reverse strategy to overcome these limitations, which is based on the action of receptors with appropriate characteristics for TDD, but without a priori knowledge of their identity or the receptor’s natural ligand (unbiased). A phage display library devoid of normal tissue-binding clones was processed through a unique, biopanning protocol, which included wild-type and drug-resistant mPrC and normal prostate cells. This led to the discovery of a pool of mPrC-specific 7-mer cyclic peptides that were subsequently subjected to functional tests, including cell uptake kinetics, internalization pathways, selectivity for patient PrC cells, chemo- and biostability, efficient conjugation to a cytotoxin, and the ability of the resultant peptide–drug conjugates (PDCs) to kill target cells in vitro and in vivo selectively. The peptides were shown not to bind to PSMA. Detailed studies on one of the peptides (P10) demonstrated that it is endocytosed by mPrC cells via a clathrin and dynamin-independent, ARF6-dependent pathway and specifically targets PrC tumors in mice. In vitro, PDCs incorporating P10 selectively killed wild-type and drug-resistant PrC cell lines as well as patient PrC cells but not normal prostate tissue cells, while in vivo, the PDCs halted the growth of PrC tumors in a xenograft model. This unique pool of peptides fulfills the functional characteristics required for effective detection and TDD inside mPrC cells. The protocol described can be easily adapted to isolate peptides for targeting other types of cancer cells.

## 2. Materials and Methods

### 2.1. Cells, Kits, and Reagents

Cell lines were obtained from the ATCC (Biological Industries, Beit Haemek, Israel). The mPrC cell lines PC-3 (RRID:CVCL_0035), LNCaP (RRID:CVCL_0395), DU-145, (RRID:CVCL_0105), and 22RV1 (RRID:CVCL_1045), and human epithelial HEK 293T/17 cells (RRID:CVCL_1926), were grown in RPMI 1640 medium (Satorius, Beit Haemek, Israel) supplemented with 10% fetal bovine serum (FBS) (Thermo Fisher, Rhenium, Modiin, Israel), 3 mM l-glutamine, and penicillin–streptomycin solution (Satorius). The medium for LNCaP cells also contained 1 µM of insulin and 1 nM of testosterone (Sigma-Aldrich, Rehovot, Israel). RPWE-1 cells derived from normal human prostate epithelium were grown in KSFM supplemented with 0.05 mg/mL of bovine pituitary extract (BPE), 5 ng/mL of EGF, and penicillin–streptomycin solution. Peripheral blood mononuclear cells (PBMCs) were obtained with consent from a healthy donor and used immediately. Normal prostate and prostate cancer biopsy tissues were obtained from the Rabin Medical Center (Beilinson campus) after approval of the study by the Rabin Medical Center’s institutional review board (0176-20-RMC). Normal tissue was obtained from consenting donors undergoing transurethral resection of the prostate (TURP) for benign prostatic obstruction, while prostate tumor biopsies were obtained from tissue resections from patients undergoing radical prostatectomy. On resection, the tissues were immediately placed in KSFM supplemented with 2.5 µg/mL of EGF, 25 µg/mL of BPE, 5% FBS, 1% penicillin-streptomycin solution, 1% amphotericin B solution, 250 mg/mL, and 10 mM HEPES, transported to the laboratory where they were promptly centrifuged, resuspended in medium without FBS and cultured on collagen type I-coated 6-well plates (Greiner, Danyel Biotech, Rehovot, Israel). All cells were maintained at 37 °C in air containing 5% CO_2_. A sample of normal human blood plasma was obtained from the Assuta Medical Center, Ramat Hachayal, Tel Aviv, Israel, as a part of an experiment unrelated to the current study.

Mouse liver was obtained from a healthy male mouse sacrificed as part of an unrelated study approved by the Ariel University Ethics Committee. The organ was washed in cold 0.01 M PBS, pH 7.4, transferred to a test tube containing 10 mL of Tris-HCl solution, pH 7.4, and homogenized for 4 min on ice using a Potter–Elvehjem glass tissue grinder. The mixture was centrifuged at 28,500× *g* and 4 °C for 20 min, after which the supernatant was collected. The homogenate was used immediately for stability testing or aliquoted and stored at −80 °C until use.

The phage display peptide library (Ph.D.-C7C, New England Biolabs, Ipswich, MA, USA) contained approximately 50 copies each of 10^8^ pfu/μL. Each phage clone displayed a unique 7-mer cyclic peptide. The kit was used according to the manufacturer’s instructions. The 7-mer cyclic library was chosen for several reasons. (a) Structural stability: Cyclic configuration improves resistance to enzymatic degradation compared to linear peptides [18,19]. (b) Selectivity: Short cyclic peptides exhibit enhanced selectivity as ligands due to constrained conformation reducing structural flexibility, promoting precise target binding [20]. (c) Higher binding affinity/selectivity via optimized orientation of functional groups and increased interaction surface area [21].

Chemicals, drugs, and reagents for peptide conjugation and other procedures were purchased from Sigma-Aldrich (Rehovot, Israel), Holland Moran (Yehud, Israel), S.L. Moran (Jerusalem, Israel), Hylabs (Rehovot, Israel) or Biolab (Jerusalem, Israel).

### 2.2. Development of Drug-Resistant Cell Lines

PrC cell lines resistant to Estramustine (ESM) were developed as the clinical use of this drug induces significant side effects and drug resistance [13,14]. The procedure of Mohr was used [15] as described in the Supplementary Information.

### 2.3. Isolation and Validation of mPrC Cell Internalizing Phage

**Figure 1:**
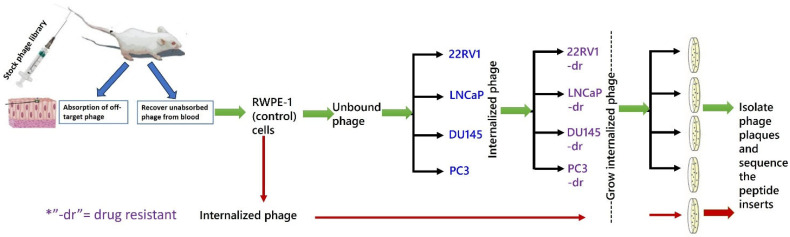
Schematic workflow for isolating phage clones specifically internalized by either a normal prostate epithelial cell line (RWPE-1) or malignant prostate cancer (mPrC) cell lines PC3, 22Rv1, DU145, or LNCap. In some cases, the isolated phage also targeted the corresponding mPrC drug-resistant cell line.

The protocol for phage selection is shown in Figure 1. Briefly, the phage library was injected into a healthy male BALB/c mouse to remove phage clones displaying peptides with affinity for normal tissue or blood cells or soluble blood components [16]. After 24 h, peripheral blood was collected, and the phages were recovered. This “absorbed” phage library was exposed to control RWPE-1 cells. Unbound and internalized phages were recovered separately, and the process was repeated. The non-binding phages were then exposed for 1 h to microplate wells seeded overnight with either PC-3, DU-145, 22Rv1, or LNCaP cells. The cells were washed in PBS, and membrane-bound phages were eluted by the addition of 400 μL of 0.2 M glycine-HCl, pH 2.2/1 mg/mL of BSA for 10 min. The solution was transferred to a sterile tube and neutralized by the addition of 65 μL 1 M Tris-HCl, pH 9.1. Four hundred microliters (400 µi) of PBS were added to the cells, which were then ruptured by freezing (−20 °C) and thawing (37 °C) (three cycles). The mixture was centrifuged at 3400× *g* for 2 min, and the supernatants containing the internalized phage were exposed to their corresponding drug-resistant cell lines. Phage internalized by these cells were recovered and reprocessed through their respective drug-resistant cell lines two more times. Phages that were bound to or internalized by RWPE-1 cells were also collected and served as phage pool internalized by non-cancerous prostate cells. All recovered phages were titrated according to the library manufacturer’s protocol. From the bacterial lawn containing a phage dilution that produced approximately 200 blue (library phage) plaques, 15–20 plaques were individually collected into separate 15 mL tubes, amplified, recovered by PEG precipitation, and their DNA was isolated. Using kit primers, the region containing the peptide insert was Sanger-sequenced, and the peptide amino acid sequence was deduced.

From the total list of phage-displayed peptide sequences, those also displayed by the RWPE-1-internalized phage were removed. The remaining mPrC clones were validated for PrC specificity by re-exposing them to RWPE-1, PC-3, DU-145, 22Rv1, or LNCaP cells for 1 h for 24 h. The internalized phages were recovered, and their titers were determined. The peptides displayed by the phage clones with the highest mPrC cell-to-RPWE-1 internalization titer ratio were synthesized and coupled to fluorescein isothiocyanate (FITC) (Pepmic Ltd., Suzhou, China) for flow cytometry analysis.

To validate the specificity of peptide binding, cells were seeded overnight, washed with PBS, and incubated for 15–60 min at 37 °C or 4 °C with 0–50 µM of peptide–FITC conjugates dissolved in RPMI/2% FBS. The cells were washed twice with PBS, trypsinized, and centrifuged at 340× *g* for 5 min. The pellet was resuspended in PBS and analyzed for FITC staining by flow cytometry, and the data were analyzed with FlowJo 10 software. Cells were first gated on a side scatter height (SSC-H) vs. side scatter area (SSC-A) plot to exclude doublets, as well as on a forward scatter area (FSC-A) and SSC-A plot to exclude small debris and cell aggregates. FITC-stained cells could then be compared to the unstained control. To negate the possibility of nonspecific FITC binding, competitive binding between FITC-labeled and unlabeled peptides was performed. PC3 and DU145 cells were seeded overnight, washed twice with PBS, and then exposed to various concentrations of unlabeled peptide in culture medium for 5 min. FITC–peptide conjugate was added at a constant concentration for 1 min, after which the cells were washed twice with PBS, trypsinized, and analyzed by flow cytometry.

#### Binding of Peptides to Normal Prostate and PrC Patient Cancer Cells

Biopsies of prostate tumor tissues and nonmalignant tissues were obtained from patients undergoing radical prostatectomy or TURP, as described above. Fresh tissue was transferred to Ariel University within 3 h, finely minced with a sterile blade, and then cultured on collagen-coated 6-well plates until the cells were attached to the well. The medium was changed twice a week. The cells used for the experiments were from passages 2–5. Passaged cells were seeded into 2 wells of 6-well plates for 24 h and then washed with PBS. One well received only medium, while the second well was exposed to 20 µM of FITC–peptide in full medium for 20 min. The cells were washed twice with PBS, trypsinized, centrifuged at 400× *g* for 5 min at 4 °C, resuspended in PBS, and mixed with 1:1 *v*/*v* fixation buffer (Invitrogen™, Rhenium, Modiin, Israel), prewarmed to 37 °C. After incubation for 10 min at 37 °C and washing in PBS, the cells were permeabilized in Perm buffer (BD™ PhoSFLOW, Biosciences, Ceasaria, Israel) for 15 min on ice, centrifuged, and resuspended in PBS. Alexa Fluor^®^ 647-conjugated anti-AMACR (alpha-methylacyl-CoA racemase) (P504S, Santa Cruz Biotechnology, Enco, Petah Tikva, Israel) was added to distinguish cancerous from normal cells. AMACR is a mitochondrial and peroxisomal enzyme that is overexpressed in prostate cancer [17]. The cells were incubated on ice in the dark for 30 min, washed twice, resuspended in PBS, and analyzed by flow cytometry.

### 2.4. Analysis of Peptide Function

#### 2.4.1. Influence of Cell Cycle on Peptide Binding

PrC cells were seeded for 24 h, washed with PBS, and exposed to 20 µM of FITC–peptide in complete medium for 60 min. The cells were washed, trypsinized, fixed in 66% ethanol on ice for 2 h, centrifuged at 500× *g* for 5 min, washed in cold PBS, and centrifuged again. The cells were resuspended and analyzed by flow cytometry for DNA content using the Propidium Iodide Flow Cytometry Kit (ab139418), (Abcam, Zotal, Tel Aviv, Israel).

#### 2.4.2. Internalization of mPrC Peptides

RWPE-1, PC3, LNCaP, 22Rv1, and DU145 cells were seeded onto 8-well MilliCell^®^ EZ slides (Millipore, Mercury, Rosh Hain, Israel) at 2.5 × 10^4^ cells/mL and cultured for 24 h. The cells were washed twice with PBS, and then cultured in fresh RPMI containing 2% FBS, and 10 µM of FITC-peptide was added for 5–60 min. The cells were washed twice with PBS, fixed with 4% PFA (pH = 7.4) for 20 min at RT in the dark, washed with PBS 3 times, stained with DAPI (VECTOR laboratories, Inc., Zotal, Tel Aviv, Israel), and imaged with an inverted Zeiss Axio-observer Z1 confocal microscope. The color channels were merged with ImageJ 1.53a software (RRID:SCR_003070).

To measure uptake kinetics, PC3 cells were seeded at a density of 5 × 10^5^/200 µL onto the well of a glass slide (Millipore Millicell^®^ EZ Slide, Mercury, Rosh Hain, Israel). The medium was replaced with RPMI 1640 medium containing 20 µM of P10–FITC and Hoechst 33342 to stain the DNA. After 20 min at 4 °C, the cells were washed with cold PBS and viewed by live microscopy (Olympus, Tokyo, Japan) at 37 °C in an environment containing 5% CO_2_. To accurately assess the kinetics of Pr10 uptake, intracellular fluorescence images were collected for the same group of cells every 10 min for 60 min. ImageJ 1.53a software (Fiji Win64) was used for immunofluorescence intensity analysis.

To visualize intracellular peptide localization, PC3 cells were seeded at a density of 5 × 10^5^/200 µL into the wells of a glass slide (Millipore Millicell^®^ EZ Slide). The following day, the cells were transduced at a confluence of 70% with 3 μL of Cell Light^™^ Lysosome-RFP reagent (BacMam 2.0, Rhenium, Modiin, Israel) by incubation for 16 h at 37 °C. Subsequently, the medium was replaced with RPMI 1640 medium without FBS but containing 20 µM of P10–FITC, and the cells were incubated at 37 °C in an environment containing 5% CO_2_. After each incubation, the medium was removed, and the cells were washed with PBS/0.05% Tween 20 and then with glycine HCl (0.1 M, pH = 2.2) for 10 min at room temperature. The cells were fixed in 4% paraformaldehyde for 10 min, washed with PBS, and stained with mounting medium containing DAPI (Vectashield). Fluorescence images were collected with a confocal microscope (ZEISS LSM700) using CellLight Lysosome-RFP (555/584 nm), DAPI, and PrC–FITC. ImageJ2 software (Fiji Win64) was used for immunofluorescence analysis.

#### 2.4.3. Peptide Endocytic Pathways

Preliminary experiments with chemical inhibitors of endocytic pathways (Dynasore, Pitstop, and MβCD) indicated that the uptake of P10 or P11 did not involve the caveolae, CIE, CDE, or DDE pathways (Figure S6). To investigate peptide uptake more precisely, single siRNAs were used to knockdown specific proteins associated with different endocytic pathways: Dynamin 2 (si-Dyn2 5′-CCCUCAAGGAGGCGCUCA-3′), ARF-6 (si-ARF-6 5′-AGGACUUUACCAAGAGCUGUU-3′), GRAF-1 (si-GRAF-1 5′-UUUGAAACUGGUACAUCAUGAGUGGUU-3′), or the control protein GAPDH (si-GAPDH 5′-UGGUUUACAUGUUCCAACA-3′) (Dharmacon, Abu-Gosh, Israel). In preliminary experiments, the maximum knockdown of protein expression was achieved by transfecting cells at 37 °C for 72 h with 25 nM of siRNA using the TransIT–X2^®^ Dynamic Delivery System (Mirus Bio, Tel Aviv, Israel) according to the manufacturer’s protocol. β-Actin was used as a loading control. The immunoblot band density was quantified using ImageJ2 software, and the relative ratios of specific siRNA/control siRNA-treated cells were calculated. Figure S7 shows that the maximum expression knockdown reached 60% for Dynamin 2 and 95% for ARF6 and GRAF1.

PC3 cells were plated overnight at a density of 5 × 10^5^ cells/mL, and replicate cultures were transfected as described above and cultured for an additional 72 h. Then, 100 µL of 5, 10, 15, or 20 µg/mL of P10- or P11-FITC; 5, 10, 15, or 20 µg/mL of Transferrin-Alexa Fluor; or 4, 8, or 10 µg/mL of fluorescent Cholera toxin B was added to separate the wells of the PC3-knockdown cultures. The cells were divided into two aliquots. One sample was processed for confocal microscopy as previously described. The second aliquot was washed in ice-cold PBS and collected on ice by scraping it into lysis buffer (Cytobuster^™^) containing a protease inhibitor cocktail. Lysates were centrifuged at 13,000× *g* (4 °C) for 10 min, and the supernatant was stored at −20 °C or processed immediately. For processing, 1 Bolt^™^ LDS sample buffer (4×) was added to the samples. The mixture was heated for 10 min at 70 °C, separated by SDS-PAGE (Invitrogen^™^), and transferred to PVDF membranes which were blocked, probed with goat antibodies to Dynamin 2, ARF-6, GRAF-1, or β- actin followed by HRP-conjugated rabbit anti-goat antibodies, and then illuminated by the addition of Luminata^™^ Crescendo Western HRP substrate (Millipore, Burlington, MA, USA), using a Life Technologies blotting apparatus (Thermo Fisher). The membranes were photographed (ImageQuant^™^ LAS 4000 Mini) and analyzed using ImageJ2 software (Fiji Win64).

### 2.5. Analysis of PrC10 Binding to PSMA

PC3 cells were seeded for 24–48 h, washed with PBS, and recultured for 10 min in fresh medium containing a 1/100 or 1/1000 dilution of rabbit anti-human PSMA or non-relevant rabbit IgG antibody. The cells were washed with PBS and incubated in fresh medium supplemented with 10 µM of PrC10-FITC for 20 min. Following incubation, the cells were washed twice with PBS, gently scraped from the wells, and resuspended in 1% BSA. The cell suspension was immediately analyzed by a flow cytometer to measure the fluorescence intensity of PrC10-FITC.

### 2.6. PDC Synthesis and Stability

The peptide showing the highest specificity for mPrC cells (P10) was conjugated to CPT and SN-38, the active component of the FDA-approved drug irinotecan as previously reported [16,18] and characterized as described in Supplementary Information (Schemes S1–S3, Figures S8, S9, and S11).

### 2.7. Cell Cytotoxicity of Free Drugs and PDCs

To test the cytotoxicity of free and P10-conjugated CPT, cells were seeded onto 96-well microplates (Nunc) at 5 × 10^4^ cells/mL for 24 h. The medium was refreshed either alone (control) or supplemented with increasing drug concentrations (0.0625–10 µM of CPT, or 0–50 µM of PDC), and the cells were cultured for 24 h. To test the cytotoxicity of free or conjugated SN-38, cells were cultured for 6 h with increasing drug doses. The cells were then washed in PBS and recultured in drug-free medium for 72 h. The cellular metabolism of cells following treatment, as determined with an XTT Cell Proliferation Kit (Satorius, Beit Haemek, Israel), was used as a measure of cell viability. Absorbances were measured with a TECAN Infinite M200 ELISA reader (Tecan Life Sciences, Mannedorf, Switzerland). The specific absorbance readings at 680 nm (nonspecific measurements) minus the readings at 480 nm were used to calculate the % growth inhibition (GI) of the surviving cells exposed to the drug compared to that of the surviving cells not exposed to the drug.

### 2.8. In Vivo Tumor Targeting

All animal experiments were performed in accordance with standard procedures for laboratory animals approved by the Institutional Animal Care and Use Committee of Ariel University (#AU-IL-2401-104). Seven 8-week-old male athymic, nu/nu mice were inoculated subcutaneously with 1.5 × 10^6^ PC3 cells suspended in Matrigel. On day 12 post-injection, 3 mg/kg of P-10-Cy5 peptide conjugate in PBS was administered via tail vein injection to five mice, while two mice received only vehicle. After 48 h, the mice were euthanized via CO_2_ inhalation, and major organs were collected, placed on Petri dishes, and fluorescence imaging taken using the IVIS Spectrum CT system (PerkinElmer, Waltham, MA, USA) (excitation filter: 640 nm; emission filter: 680 nm; exposure time: 0.5 s; binning: medium (8)).

### 2.9. Xenograft Study to Evaluate Anti-Tumor Effect of PrC10-SN38

As described above, a xenograft tumor model was established by injecting mice with PC3 cells. Tumor growth was monitored regularly, and mice were randomized into one of four groups (n = 5 per group) when their tumor volume reached approximately 140 mm^3^ (approx. day 16 post-injection) so as to ensure the equal distribution of tumor burden among the groups. The mice were treated twice weekly for four weeks via tail vein injection as follows. Group 1 (Control) received vehicle only; Group 2 (free SN-38) received SN38 at a dose of 5 mg/kg; Group 3 (PDC 10 mg/kg) received PrC10-SN38 at a dose of 10 mg/kg; Group 4 (PDC 20 mg/kg) received PrC10-SN38 at a dose of 20 mg/kg. Tumor volume was measured regularly until day 44 from the initiation of treatment, after which all mice were euthanized via CO_2_ inhalation.

### 2.10. Statistics

The statistical significance of differences between groups was determined using Student’s *t* test. A *p* value ≤ 0.05 was considered to indicate statistical significance. A comparison of the IC_50_ values to the specified values was performed with the Wilcoxon signed-rank test. The percentage of positive antibody binding in flow cytometry was calculated with the Overton cumulative histogram subtraction algorithm in FlowJo software.

## 3. Results

### 3.1. Drug-Resistant Cell Lines

The dose–response curves of the PrC cell lines to ESM are shown in Figure S2. The IC50s for ESM ranged from 1.4 to 1.75 µM. ESM-resistant lines developed following more than 6 months of exposure to the drug. These cells also demonstrated multidrug resistance to both Camptothecin (CPR) and docetaxel (Figures S1 and S2, and Table S1).

### 3.2. Selection and Validation of PrC-Specific Peptides

Using our phage selection protocol (Figure 1), 88 phage clones were recovered, and their peptide inserts were sequenced. Of these, 20 (22.7%) did not internalize into RWPE-1 negative control cells. These clones were re-exposed to RWPE-1, PC-3, DU-145, and 22Rv1 cells, after which the internalized phages were recovered and titrated. For each clone, a “Phage PrC specificity” (PPS) index was obtained by calculating the ratio of the internalized phage titer for a given PrC cell line to the internalized phage titer for RWPE-1 cells. Six of these clones were chosen for further study (Table S2).

**Figure 2:**
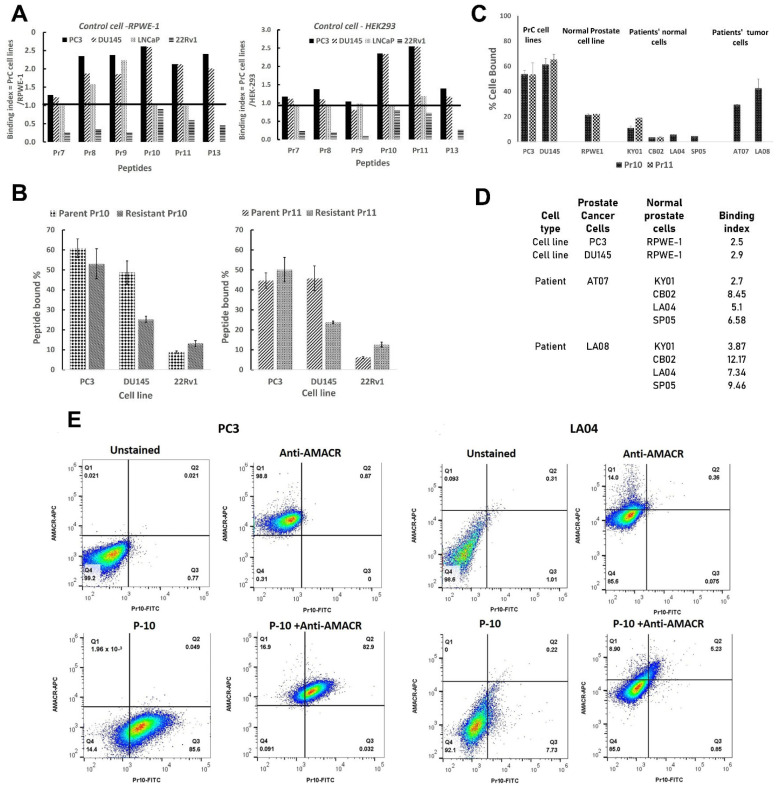
Peptides P10 and P11 bind PrC drug-resistant cell lines and cancer cells from patients with PrC. (**A**) Determination of Peptide PrC Specific Binding Index (PSBI) of 6 PrC–peptide–FITC conjugates for prostate cell lines versus two control cell lines, RPWE-1 and HEK-293. In total, 10 µm of each peptide–FITC conjugate was incubated with each cell line, and the % positive cells were determined by flow cytometry. The horizontal bar represents the ratio of binding to the respective control cell lines. A parametric and independent 1-tailed Student *t*-test was used to compare the difference in the binding. A comparison of PrC cell lines to normal lines gave *p* = 0.037 and 0.014 for Pr10 and Pr11, respectively. If the comparison is only for PC3 and DU145 lines vs. normal cell lines, the *p*-values are <0.0001 and 0.00015, respectively. (**B**) A comparison of peptides Pr10 (left) and Pr11 (right) binding to parent PrC cell lines (PC3, DU145, and 22Rv1) vs. their resistant cell lines. Exposure for 10 µM of peptide for 60 min. Using the 1-tailed student *t*-test for independent samples, there were no significant differences between the parental and resistant cells at each time-point (*p* = 0.14–0.31). (**C**): Binding of Pr10 and 11 to prostate cancer cell lines, patient cancer cells. (**A**) Peptides Pr10 and Pr11 binding to PC3 and DU145 cell lines, donor cells, and RPWE1. Due to a lack of cells, LA04 and SP05 were only exposed for Pr10. (**C**) Binding indices of prostate cancer cells versus normal prostate cells. (**D**) Peptide Binding Index of prostate cancer vs. normal prostate cells. (**E**) Four-quadrant flow cytometry data demonstrating the double-staining of PC3 or normal prostate cells (LA04) for P10-FITC and an Alexa Fluor^®^ 647 fluorescinated antibody to the enzyme AMACR, which is overexpressed in prostate cancer. Unstained cells (top left of PC-3 and LA04) were used to gate for background fluorescence. Cells were stained with either Alexa Fluor^®^ 647 fluorescinated Anti-AMACR antibody alone (top right for each cell), P10-FITC alone (bottom left for each cell), or Anti-AMACR and p10-FITC together (bottom right for each cell). The % of positive cells in each quadrant is shown.

The peptides displayed by these phage clones were synthesized together with the fluorescent marker FITC, and their binding to PC and control cell lines was determined by flow cytometry. From these results, a peptide-specific binding index (PSBI) was assigned to each peptide by determining the ratio of peptide–FITC (at 10 µM) binding to each PC line compared to that of the control cell lines. Figure 2A shows that only the peptides P10 and P11 produced a statistically significant PBI for all the PC cell lines (*p* = 0.037 and *p* = 0.0144, respectively). The binding of these two peptides to PC3 and DU145 cells was concentration, time, and temperature-dependent (Figure S3) and occurred only in the G2/M phase of the cell cycle (Figure S4). P10- or P11-FITC conjugates also bound to the multidrug-resistant cell lines (Figure 2B). Although their binding to drug-resistant DU145 cells was lower than that to the parent line, this difference was not statistically significant. As expected, binding to 22Rv1 cells was lower than that to the other two lines.

Normal prostate tissue samples were obtained from five patients undergoing a TURP procedure, and primary cultures from four of them (coded KY01, CB02, LA04, and SP05) were successfully established after 4–6 weeks of culture. Prostate cancer tissue samples were obtained from three patients who underwent radical prostatectomy, and slow-growing primary continuous cultures were established from two of them (coded AT07 and LA08). Figure 2C shows that P10 and P11 both distinguished between the PrC lines and the normal cell line RPWE-1 by a factor of 2.5–3.0. More importantly, P10 distinguished between PrC cells from patient biopsies and normal prostate cells by a factor between 2.7 and 12.2 (Figure 2D). Unfortunately, there were insufficient patient cancer cells to test the binding of P11.

To further test the tumor cell binding specificity of P10 for PC3, and LA04 cells, they were double-stained with P10-FITC and an anti-AMACR-Alexa Fluor^®^ 647 conjugated antibody (Figure 2E and Figure S5). Of the 99.8% AMACR+ PC3 cells, 82.9% were also P-10+, resulting in 83% positive concordance. Interestingly, while 93.9% and 86% of LA04 cells were negative for AMACR and P-10, respectively (91.6% negative concordance), 14.13% of LA04 cells were AMACR+, while only 6.08% were P-10+. These results demonstrate that P10 is a specific PrC-binding peptide and may be more accurate in detecting prostate cancer cells than AMACR. Unfortunately, prostate tumor cells from patients SP07 and LA08 could not be maintained to allow the analysis of the binding of the peptide and AMACR marker.

### 3.3. P10 and P11 Internalize into the Cytosol of PrC Cells

**Figure 3:**
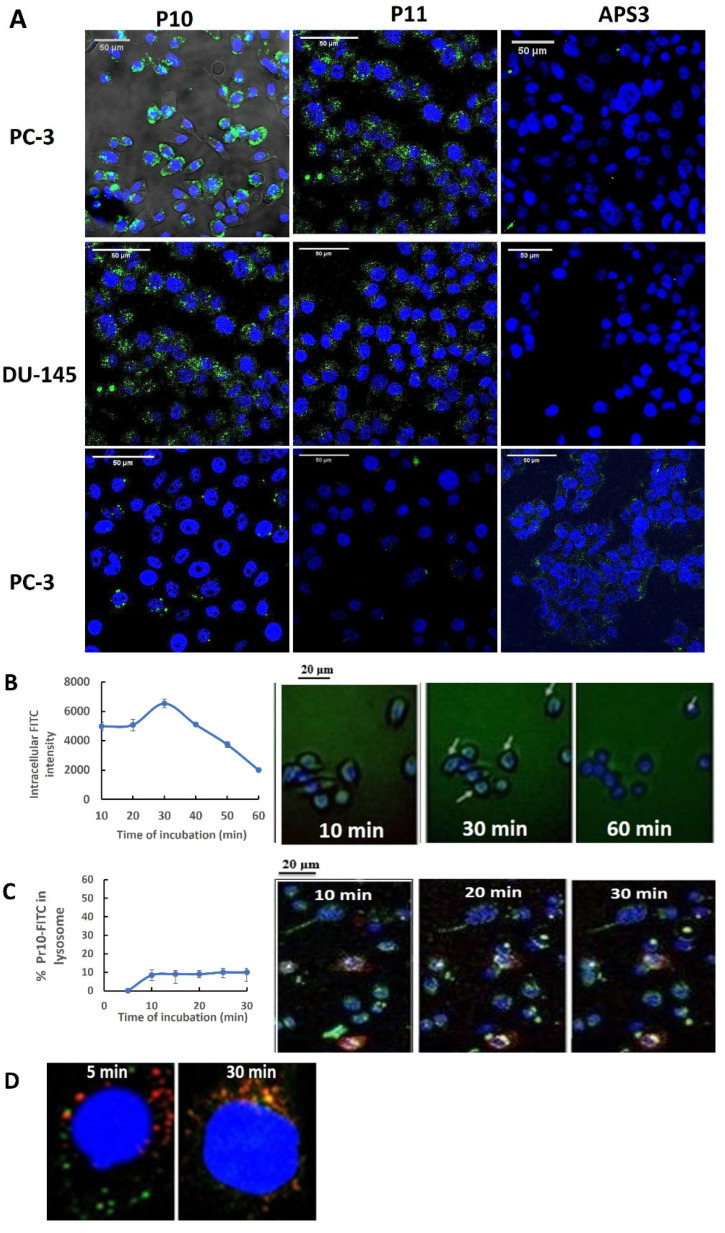
P10 and P11 internalize into the PrC cell cytoplasm. (**A**) Confocal microscope images of PC3 and DU145 cell lines exposed to 10 µM of Pr10 peptide for 5 min. The images were captured with a fluorescent DAPI filter with white light to highlight the cell contour and FITC filter for the peptide. Color channels were composited in ImageJ software. Magnification ×40. (**B**) Live-cell microscopy of P10-FITC internalization into the same group of PC3 cells over time (**right**). Qualitative assessment of fluorescence intensity following incubation of PC3 cells for 10 min, 20 min, 30 min, 40 min, 50 min, or 60 min with P10-FITC and Hoechst 33342 (excitation wavelength 460–490 nm, emission 470 nm); ×20 objective magnification (**left**). (**C**) Quantitative analysis of live-cell microscopic images from A, analyzed by calculating the incremental change in intracellular FITC intensity over each period of incubation time, beginning with the 10 min incubation image. The data points represent the mean and standard deviation of fluorescence intensities of at least 5 cells in the field analyzed. (**D**) Intracellular localization of endocytosed P10 in PC3 cells. Live-cell microscopic tracking of P10-FITC at time-points of up to 30 min following conjugate uptake. Cells were incubated first with a red fluorescent lysosome marker, then with DAPI to label cell nuclei, and finally with P10-FITC. The localization of P10-FITC in lysosomes in representative cells after 5 and 60 min incubations is indicated by yellow fluorescence. Magnification, ×20.

The internalization of P10 and P11 into cells was tracked by confocal microscopy. Figure 3A shows that the intercellular staining by P10-FITC was strongest for PC3 and DU145 cells and stronger than that by P11-FITC. In contrast, there was no uptake of the non-relevant 7-mer cyclic peptide APS into these cells. FITC did not colocalize with the DAPI marker, suggesting that the peptides did not enter the nucleus.

The time-dependent internalization of P10 into parent and drug-resistant lines was determined using ImageJ software to quantify the fluorescence intensity in cells incubated with peptide–FITC for 10, 30, or 60 min at 37 °C. The area of green peptide–FITC fluorescence was divided by the area of blue DAPI fluorescence to normalize the quantity of green signals per cell. Cells in four microscope fields were analyzed for each incubation time and cell line (150 cells, range of 110–191 cells). Figure S3 (bottom) shows that for drug-resistant PC3 cells, the accumulation of P10 appeared to be elevated above that of the parent line by 60 min. For DU145 cells, the uptake into resistant cells appeared to remain slower than that for the parent line. However, these differences were not statistically significant.

The kinetics of Pr10 uptake were assessed using live-cell microscopy by focusing on one group of cells over 60 min, as shown in Figure 3B (right). Quantitative analysis indicated that fluorescence intensity peaked at 30 min (Figure 3B, left). Next, the intracellular traffic of P10 was tracked by co-labeling PC3 cells with a red fluorescent lysosome marker, then with Hoechst 33342, and finally with P10-FITC. Live microscopy captured cellular fluorescence over the following 30 min, as shown in Figure 3C, (right). The results suggested that only a minimal amount of conjugate was localized to lysosomes (approximately 10%). This result was supported by confocal images of a representative single cell, which showed that after a 30 min incubation, there was minimal overlap between the conjugate and lysosome labels (Figure 3C, left)

#### P10 Is Endocytosed via an ARF-6-Dependent Pathway and Does Not Bind to PSMA

**Figure 4:**
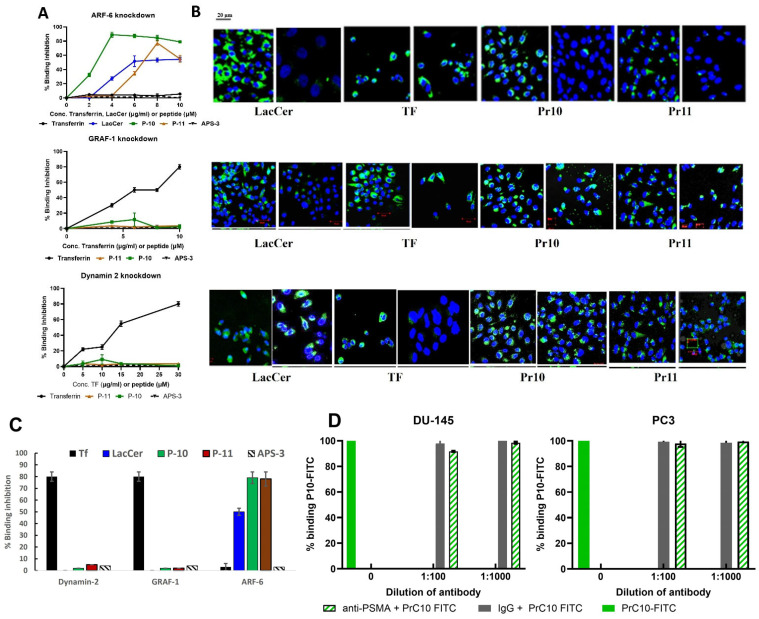
Endocytosis pathways of P10 and P11. (**A**) Flow cytometry results showing the effect of siRNA knockdown of ARF-6, Dynamin-2, or GRAF-1 on uptake of controls (Transferrin and/or LacCer) or peptide-FITC into PC3 cells. The data points represent the mean ± standard deviation of at least three repeat experiments, each one run in triplicate. Only the knockdown of ARF-6 inhibited the binding of P10 and P11. The negative control peptide APS-3 did not bind cells under any condition (**B**) Confocal microscopy images validating the effect of siRNA knockdown of ARF-6, Dynamin-2, or GRAF-1 on the uptake of controls (Transferrin and/or LacCer) or peptide–FITC into PC3 cells. Each image is a merging of separate images of DAPI and FITC. Filters used: DAPI (excitation wavelength, 460–490 nm; emission, 470 nm); FITC (excitation, 495 nm; emission, 525 nm); magnification ×40. Ruler scale = 20 μm. (**C**) Summary of flow cytometry results showing maximum siRNA-induced inhibition of binding of P-10 (Green), P-11 (Brown), and controls. (**D**) P10 does not bind to PSMA. PC3 and DU145 cells were cultured for 10 min in fresh medium containing a 1/100 or 1/1000 dilution of rabbit anti-human PSMA or non-relevant rabbit IgG antibody, then incubated in fresh medium supplemented with 10 µM of PC10-FITC for 20 min. Following washing, the cells were analyzed by a flow cytometer to measure the fluorescence intensity of PC10-FITC. The bars represent the average % binding of P10-FITC normalized to the binding in the presence of non-relevant rabbit IgG.

Based on preliminary experiments with the chemical inhibitors of endocytosis (Figure S6), siRNAs were used to knockdown the expression of specific proteins important for different endocytic pathways. Knocking down Dynamin-2 or GRAF-1 did not inhibit the endocytosis of P-10 or P-11; however, knocking down ARF-6 completely inhibited the uptake of both peptides in a dose-dependent fashion (Figure 4A,B). These results are summarized in Figure 4C.

In addition, the results of the inhibition experiments shown in Figure 4D demonstrate that P10 does not bind to the PSMA membrane protein.

### 3.4. PDC-Targeted Cytotoxicity

**Figure 5:**
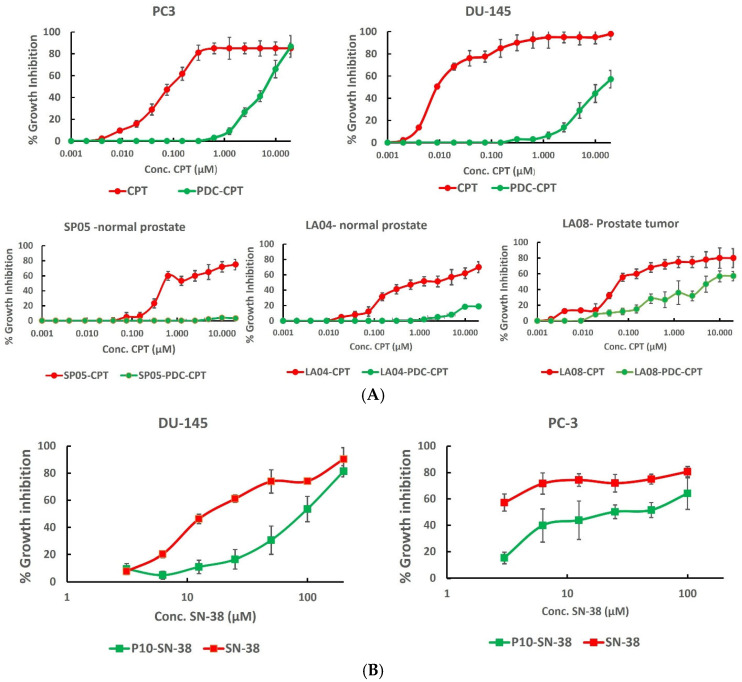
Dose–cytotoxicity curves of P10–Camptothecin and P10-SN-38 PDCs. (**A**) PrC cell lines (PC3 and DU-145) or normal prostate (SP05 and LA04) or tumor (LA08) cells derived from biopsies of prostate cancer patients were cultured for 48 h with increasing concentrations of the free CPT of P10-CPT PDC. (**B**) PC3 and DU145 cells were cultured with increasing concentrations of free SN-38 of P10-SN-38 PDC for 6. Cells were washed and recultured for 72 h. Cell viability was assessed by the XTT assay. Results are expressed as the % growth inhibition at each drug concentration compared to the no-drug control culture. The results show the mean of repeated experiments.

In light of the results above, Pr10 was chosen for conjugation to CPT and to SN-38 to produce PDCs. For details of the synthesis and characterization, see the Supplementary Information. The selective cytotoxicity of the Pr10-CPT PDC for prostate cancer cells was tested by culturing LA04, SP05, LA08, DU-145, and PC3 cells with increasing concentrations of the PDC for 48 h and measuring cellular viability. The PDC demonstrated a linear increase in cytotoxicity against both DU-145 and PC3 cells, reaching 87.6% at 20 µM for the latter cell line (Figure 5A). Cell sensitivity was higher to the free drug, which enters the cell by passive diffusion, whereas PDC endocytosis is dependent on receptor expression. This is demonstrated by the negligible activity of the PDC against the normal prostate cells SP05 and LA04. In contrast, PDC cytotoxicity against prostate cancer cells from patient LA08 reached 57% at 10 µM of PDC, representing a 3–14-fold greater selectivity of the PDC for prostate cancer cells than for normal prostate cells. Peptide P10 was also conjugated to SN-38, and the PDC effectively killed both PC3 and DU-145 cells (Figure 5B).

### 3.5. In Vivo Studies

#### 3.5.1. Tumor Targeting

**Figure 6:**
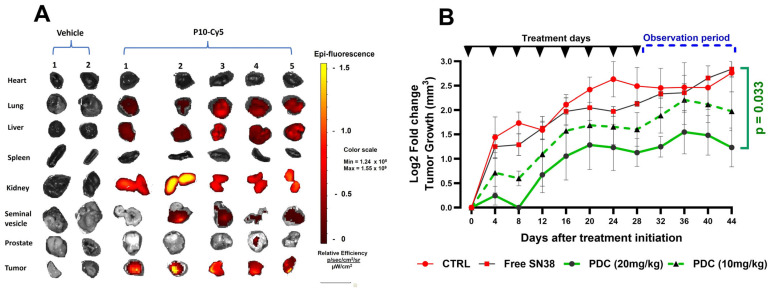
In vivo tumor targeting of P10 and anti-tumor efficacy of P10-SN-38 PDC. (**A**) Tissue distribution of PC10-Cy5 (3.33 mg/kg) in PC3 tumor-bearing athymic nude mice 48 h post-intravenous injection. Individual organs were removed and placed on Petri dishes, and their fluorescence intensity was imaged. Organs were then arranged as shown to allow a direct comparison between animals. Control tumor-bearing mice were injected with vehicle alone. The epifluorescence scale is shown. The images show a prominent accumulation of labels in the tumor mass as well as in the kidney, presumably due to filtration and excretion. (**B**) Normalized growth of prostate tumors in a xenograft mouse model. PC3 cells were injected subcutaneously into the right flank of athymic nu/nu mice. Animals were randomized into groups of 5 when their tumor volume reached approximately 140 mm^3^ (approx. day 16 post-injection). The mice were then treated twice weekly for four weeks with either vehicle (Control, Ctrl); 5 mg/kg of Free SN-38; 10 mg/kg or 20 mg/kg of P10-SN-38 PDC. Tumor volume was measured regularly until day 44 from the initiation of treatment, after which all mice were euthanized. The mean ± standard deviation of Log2 fold changes over time for each group is shown. Treatment with 20 mg/kg of PDC significantly reduced tumor volume compared to untreated control mice at day 44 (*p* = 0.033, *t* =2.253, one-tailed, unpaired *t*-test).

Cy5-labeled P10 or PBS was injected into athymic mice bearing subcutaneous PC-3 tumors. After 48 h, animals were sacrificed, the tumor and other organs were excised, photographed, and the pictures arranged as shown in Figure 6A to directly compare Cy5 distribution between the animals. An analysis of label distribution 48 h later showed significant retention in the tumor mass (Figure 6A). No label was detected in the prostate, heart, or spleen. A low level of the label accumulated in the lung tissue and seminal vesicles of some animals, but this was minimal compared to the level of labels in all the tumors. As expected, the label was detected in the tissues involved in conjugate breakdown (liver) and especially clearance (kidney).

#### 3.5.2. Anti-Tumor Efficacy

Finally, the anti-tumor efficacy of the P10-SN-38 PDC was tested in a xenograft model using PC3 cells. Figure 6B shows that during both the treatment period and the subsequent observation period, tumor progression in mice treated with 10 or 20 mg/kg of PDC was consistently lower than untreated control mice. This is in contrast to the observation that free SN-38 did not inhibit tumor growth. Indeed, from day 36, tumors in PDC-treated mice began retracting while those in the vehicle or free drug-treated mice continued to grow.

## 4. Discussion

There is an increasing interest in using peptides as drug carriers in targeted drug delivery systems, including delivery to prostate cancer cells [19], but to date, only two PDCs have received FDA approval [20]. One reason for this limited success is that most studies have selected candidate peptides based on their selective binding to tumor-associated receptors known to be overexpressed on the cancer cells [21], even though those receptors may not be optimal candidates for use in targeted drug delivery. An example of one such receptor is CD33, also known as Siglec-3, which was originally used for targeted drug delivery to Acute Myelogenous Leukemia (AML) cells [22]. A more holistic approach to selecting peptides that fulfill additional important functional requirements may result in the discovery of more effective drug carriers for targeted drug delivery to cancer cells. To accomplish this goal, we began by using a phage display peptide library preabsorbed in vivo on normal tissue to screen for peptides that internalized into PrC cell lines and PrC patient tumor cells, without a priori knowledge of the target receptor (unbiased) (Figure 1). The peptides displayed by these phages were subjected to a set of biochemical, biological, and functional in vitro and in vivo tests to ensure that they would be appropriate candidates for pre-clinical targeted drug delivery studies to effectively treat mPrC.

Several key conclusions can be drawn from our results. First, from the initial six phage clones chosen from the library screen, only two clones displayed peptides, P10 and P11, which in their free form retained significant binding above both negative control cell lines (Figure 2A). This result highlights the important point that the activity of a phage-displayed peptide does not necessarily predict the activity of the free peptide. This may be due to structural changes in the peptide when displayed as an attachment to a large protein (in the present case, to the P3 tail protein of the M13 phage) compared to the free protein, which would affect binding activity [23].

Second, it is critical to ensure that the target-positive phage clones also internalize into drug-resistant cells (Figure 2B,C). This is particularly important, as initial applications of PDC therapy will presumably not be for front-line therapy but rather for treating patients whose cancer cells are already drug-resistant.

Third, following the demonstration of target cell specificity in vitro, it is necessary to show that the candidate peptide targets tumor cells in vivo. This was demonstrated for P10 (Figure 6A). Although epifluorescence imaging of internal organs also showed an accumulation of Cy5 labels in the liver and kidney, this is presumably due to filtration and excretion.

Fourth, it is important to acquire initial information on the nature of peptide uptake and its intracellular trafficking, as this can have important implications for PDC breakdown and drug release following conjugate uptake. Little attention has been given to defining the mechanism of drug carrier uptake in drug delivery systems [12,24]. Initially, we studied the uptake of P10 and P11 by first using the chemical inhibitors of endocytic pathways, MβCD, Pitstop 2, and Dynasore. We found that Dynasore did not inhibit peptide uptake, suggesting that neither P10 nor P11 utilizes dynamin-dependent pathways (Figure 5A-left). In contrast, Pitstop 2, which inhibits both the CIE and CDE pathways, blocked P11 uptake, while MβCD, which inhibits the Caveolae + CIE pathways, blocked P10 uptake (Figure S6), suggesting that these two peptides might utilize different uptake pathways and presumably bind different receptors.

Although the chemical inhibitors provided us with general indications of the pathways used by these peptides, they could not provide sufficient detail. For this reason, siRNAs were used to knockdown the expression of specific protein components of certain endocytic pathways. These experiments confirmed that P10 and P11 do not use dynamin-dependent pathways, nor is their uptake dependent on the expression of GRAF1 (a GTPase regulator associated with focal adhesion kinase-1) (Figure 4A). These results are in line with what is known about GRAF-1, an essential component of clathrin-independent endocytic pathways, which colocalizes with dynamin during the organization of molecular networks and membrane dynamics [25]. These results also indicate that once endocytosed, the peptides are not transported to the Golgi apparatus [26,27]. In contrast, the siRNA-mediated knockdown of ARF6 completely inhibited the uptake of P10 (94%) and P11 (90%), albeit at different concentrations (Figure 4A). ARF6 (ADP-ribosylation factor 6) is involved in various functions associated with trafficking and plasma membrane protein recycling to the cell surface [28,29]. Internalization via ARF-6 is clathrin- and dynamin-independent but cholesterol-dependent [26]. ARF6 has been associated with several cell-surface receptors expressed on cancer cells, such as CD147 [30], CD44, CD98 [31], CD55, and CD59 [32]. In particular, the expression of CD55 and CD59 on PrC cells may be associated with promoting cell survival and contributing to the metastatic potential of prostate cancer cells [33]. Recently, it was shown that ARF-6 is involved in the recycling of CIE proteins (CD98 and CD147), which are rapidly removed from endosomes after entry into recycling tubules and thus do not move on to lysosomes [34]. These data may help explain the results of Figure 3D, which show, by double-label fluorescent tracking, that P10-FITC is not taken up into lysosomes. Indeed, uptake into lysosomes has been recognized as a major limiting factor in the effective delivery of nanomedicines [35].

Fifth, using the unbiased exposure of the phage library to the target cells rather than to a pre-determined receptor can lead to the discovery of peptides targeting receptors not previously recognized as potential targets. We found that our lead peptides do not bind PSMA using this approach. While this receptor is abundant on most PrC cells, radiolabeled ligands targeting it can lead to significant side effects [7,8].

The purpose of carrying out these initial functional assays was to select peptide candidates that would effectively deliver drug payloads into target cells and kill them. As peptide P10 fulfilled these functional criteria, it was selected for chemical conjugation to the Topo I inhibitor drugs CPT and SN-38 and subjected to the second testing stage. This stage initially involves the demonstration of selective in vitro target cell cytotoxicity. As predicted from the binding results (Figure 2C,D), the P10-PDCs (P1-CPT and P10-SN-38, see Supplementary) specifically killed PrC cells but not healthy prostate tissue (Figure 5). The final functional test is in vivo efficacy in a murine cancer model. Figure 6B shows that the P10-SN-38 PDC significantly halted tumor progression in a dose-dependent manner. From day 36, tumor sizes in the PDC-treated mice began to regress, while those in the control and free SN-38-treated mice continued to increase.

## 5. Conclusions

Given that current chemo- and immunotherapies for mPrC are inadequate [3], the stringent selection of peptide candidates as drug carrier candidates for TDD systems is essential. One current limitation in developing TDDs for mPrC has been the attraction to target cell surface receptors known to be overexpressed in these cells, such as PSMA. The protocol outlined in our study led to the discovery of a series of novel cyclic peptides that fulfill critical functional criteria essential for the successful detection of and TDD for mPrC. Given that their cellular uptake does not involve PSMA, they open up possibilities not only to deliver drugs through different intracellular pathways but also to identify receptors whose role in mPrC was previously unrecognized. Furthermore, the methods described constitute a generic platform strategy that can be adopted to develop PDCs with the properties necessary for effective targeted drug delivery to other types of cancer cells.

## Data Availability

Additional data will be supplied upon reasonable request.

## Supplementary Materials

The following supporting information can be downloaded at: https://www.mdpi.com/article/10.3390/pharmaceutics17070866/s1, Figure S1: Growth inhibition curves; Figure S2: Dose-response curves; Table S1: IC_50_ values; Table S2: PPS ratio for Phage clones; Figure S3: Peptides P10 and P11 binding to PC3 and DU145 cell lines and Ratio of fluorescence intensity of peptide-FITC to nucleus DAPI staining of PC3 and DU145 parent and resistant cell lines; Figure S4: The binding of P10 to PC3 and DU145 cell lines; Figure S5: Inhibition of uptake of relevant positive control, P10, P11 or a non-relevant peptide (APS-3) in the presence of increasing concentrations of chemical inhibitors of endocytic pathways Dynasore, Pitstop-2 or MβCB; Figure S6: Immunoblots of the optimal conditions for knocking down expressions; and analysis and relative ratios of specific siRNA/control siRNA treatment cells; Figure S7: CPT drug reaction with + pNPC resulting in 4-nitrophenyl carbonate derivative of CPT; Figure S8: Peptide P10 with Ahx spacer was reacted with a CPT derivative with carbonate ester site, resulting in Peptide P10-CPT PDC; Figure S9: (a) LC-MS output of the P10CPT conjugation; (b) Chemical and biological stability of the P10-CPT PDC; Scheme S1: Synthesis of Boc-SN38-PNC; Scheme S2: SPPS of P10-Ahx-Fmoc; Scheme S3: Synthesis of P10-Ahx-SN38. References [36,37,38] are cited in the supplementary materials.
