# Supplementary material for: Applying Unbiased, Functional Criteria Allows Selection of Novel Cyclic Peptides for Effective Targeted Drug Delivery to Malignant Prostate Cancer Cells
Source: Pharmaceutics. 2025 Jul 1;17(7):866. doi: 10.3390/pharmaceutics17070866 (PMC12298539; doi:10.3390/pharmaceutics17070866)
Supplement: Supplementary file 1 [file pharmaceutics-17-00866-s001.zip › pharmaceutics-3608815-supplementary.pdf]

Supplementary Information

Development of drug resistant cell lines

Cells were seeded in 3 flasks. After 24 hrs two flasks received fresh medium supplemented with the drug at 50% maximal growth inhibitory concentration (IC50) for 72 hr; the 3rd flask received fresh fresh drug-free as a control. When the cell confluency in the drug-supplemented flasks had decreased to 30–50%, the medium was changed to drug-free, and the cells were allowed to expand and were again split into 3 new flasks, two of which were exposed to a 1.5–2-fold higher concentration of the drug, while the third was retained as a control. In this way, cells were progressively exposed to increasing concentrations of the drug until they reached the concentration at which they could not recover, or, as in the case of ESM, until its aqueous solubility threshold was reached. The proliferation rates of these drug-resistant lines were then tested and compared to those of their parent lines, and the 50% inhibitory concentration (IC50) fold change compared to that of the parent cell line was calculated. The exposure of the cell lines to ESM continued up to 100 µM (the dissolution threshold). For CPT, the upper limits were 2 µM for PC3 and 22Rv1 and 0.3 µM for the DU-145 cell line. When cells surviving at these concentrations were transferred to drug-free medium, they lost their resistance to the drug within 2–4 weeks. Therefore, to maintain drug resistance, the cells were maintained in medium containing a low concentration of the drug (10% of the IC_{50}).

a) Baseline sensitivity of malignant prostate cell lines to Estramustine (ESM) and Campothecin (Cpt).

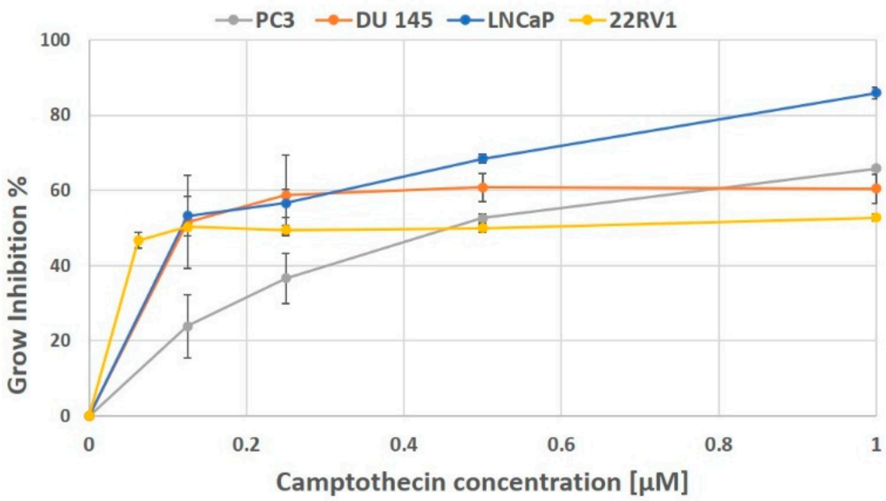

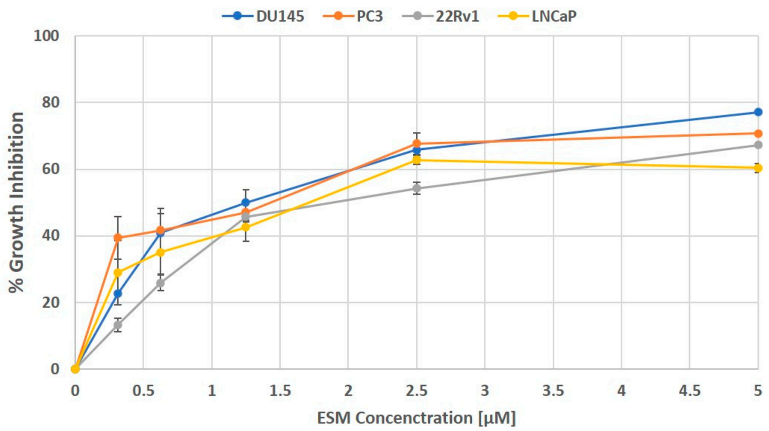

Figure S1. Growth inhibition curves of PC3, DU145, LNCap and 22RV1 malignant prostate cancer cells exposed to increasing doses of Camptothecin (upper graph) or Estramustine (lower graph).

b) The ESM-resistant cell lines developed multidrug resistance to Cpt and Docetaxel (Doc).

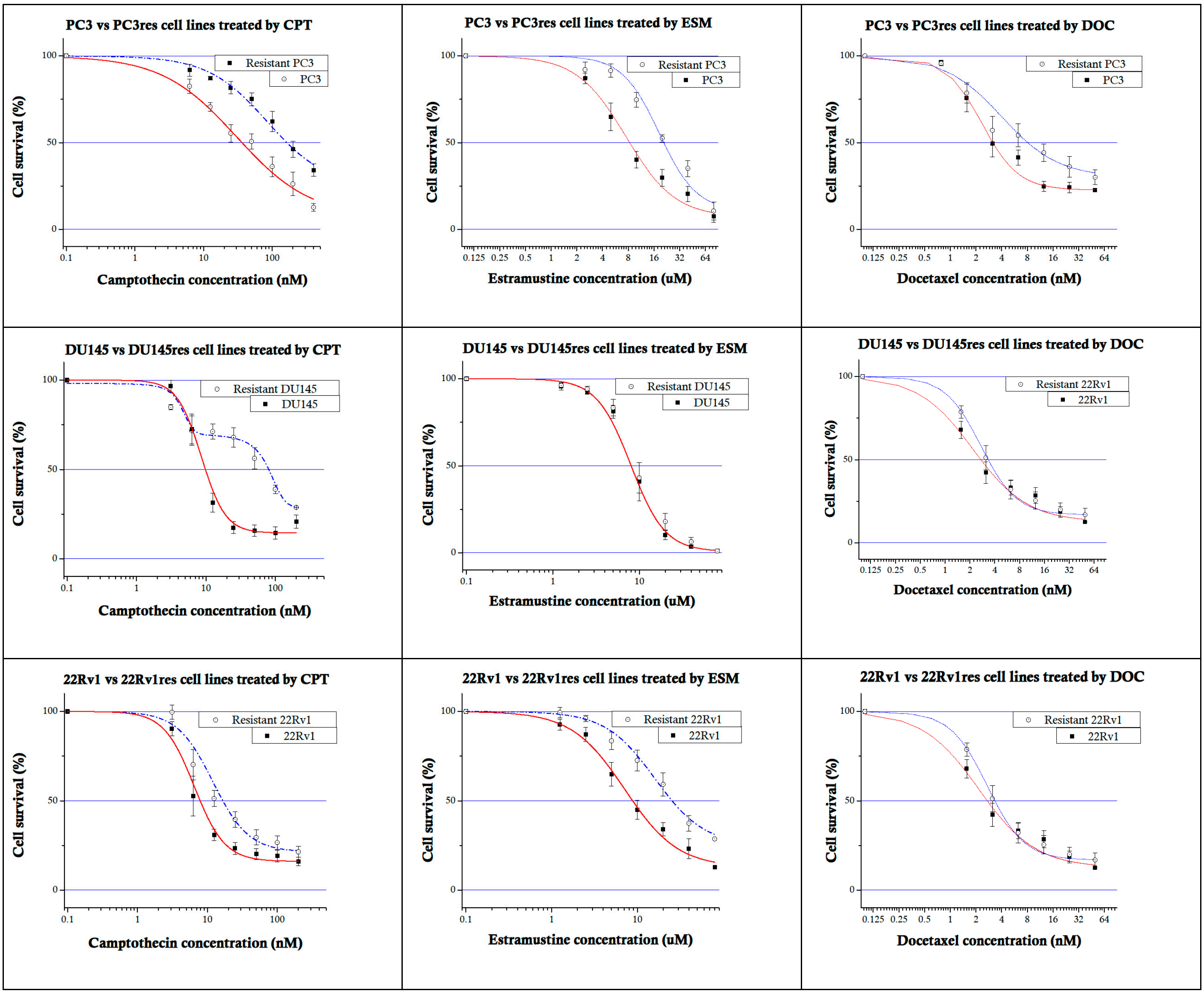

Figure S2: Dose-response curves of Estramustine-resistant prostate cancer cell lines exposed to Estramustine, Camptothecin, and Docetaxel.

Table S1: IC_{50} values for parent and Estramustine-resistant prostate cancer cell lines are also multidrug resistant to Camptothecin and Docetaxel.

	**Drugs**					
	**Estramustine**		**Camptothecin**		**Docetaxel**	
**Cell lines**	IC_{50} [µM]		IC_{50} [µM]		IC_{50} [µM]	
	Parent line	Resistant line	Parent line	Resistant line	Parent line	Resistant line
**PC3**	10	24	0.045	0.20	0.004	0.01
**DU145**	8	8	0.01	0.1	0.003	0.005
**22RV1**	8	30	0.007	0.023	0.003	0.004

Table S2: Phage clones isolated using the protocol described in Figure 1 and with a "Phage PrC specificity ratio (PPS)" * greater than 5.

**Phage clone**	**7**	**8**	**9**	**10**	**11**	**13**
**PPS ratio-PC3**	0.8	0.17	8.75	1.6	0.7	7
**PPS ratio-DU-145**	53.3	33.3	1.38	1.8	1.3	10
**PPS ratio-22V1**	0.66	1.66	1.88	5	8	1

* PPS= the ratio of the internalized phage titer for a PrC cell line / the internalized phage titer for RWPE-1 cells

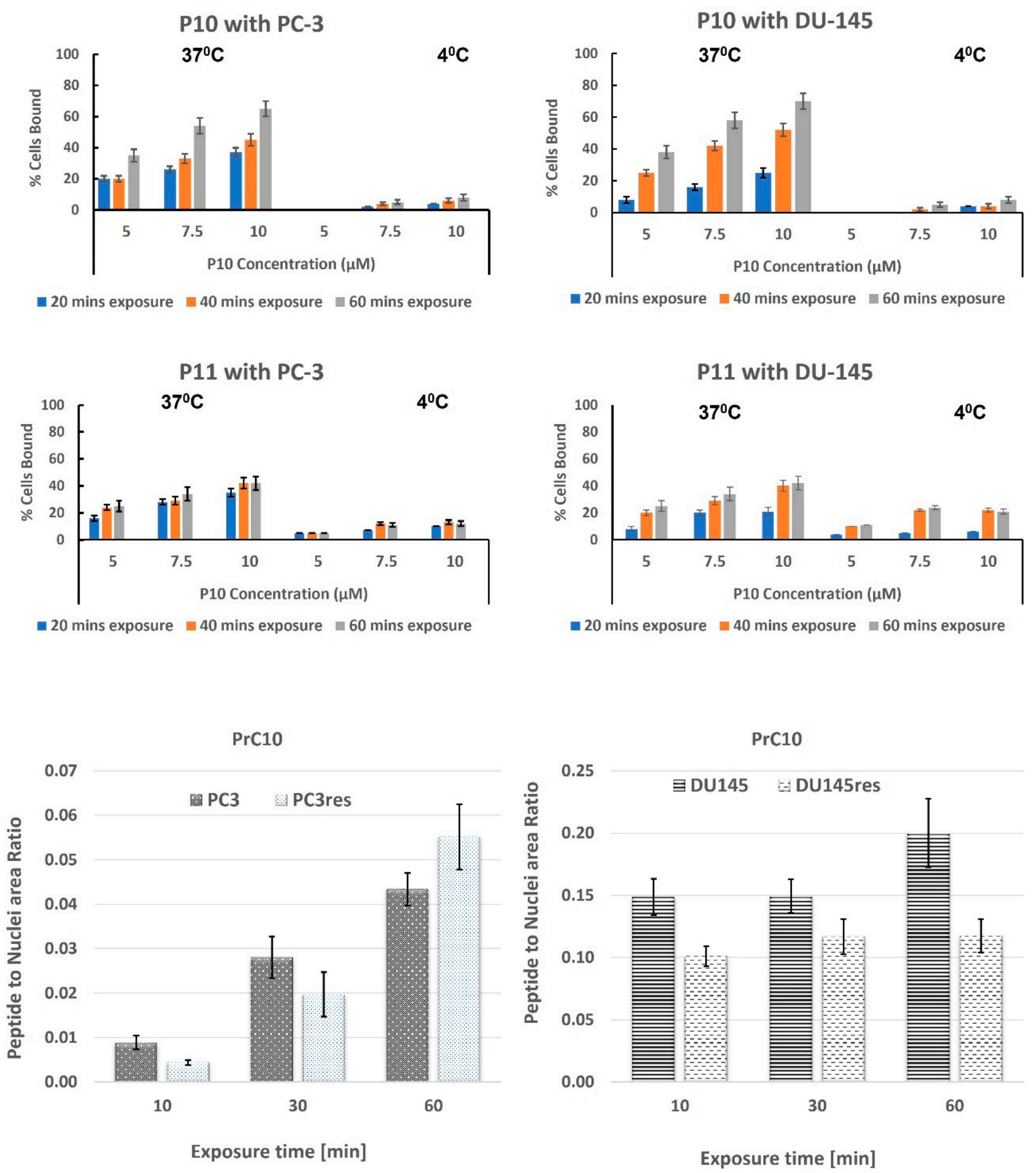

Figure S3: (top) Peptides P10 and P11 binding to PC3 and DU145 cell lines is dependent on temperature, concertation, and exposure time.

(bottom) Ratio of fluorescence intensity of peptide-FITC to nucleus DAPI staining of PC3 and DU145 parent and resistant cell lines. Cells were exposed to peptide for 10, 30 and 60 min. The data represent the mean and standard deviation of three independent experiments.

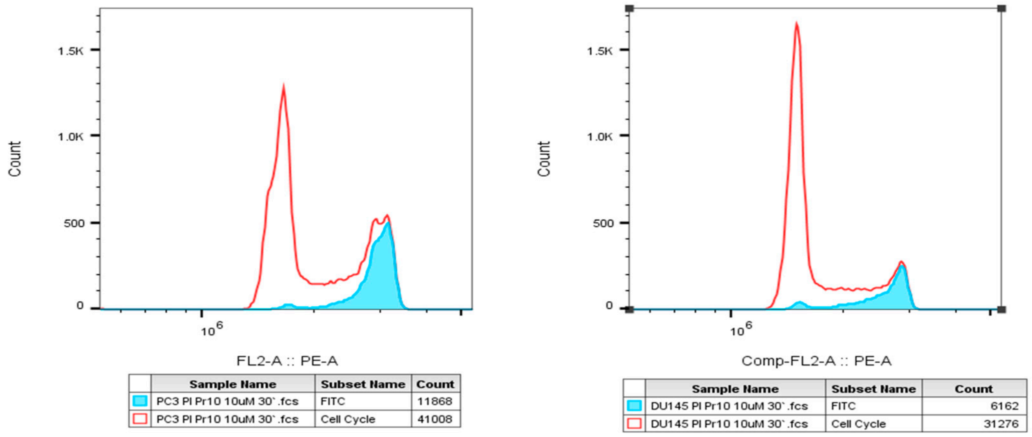

Figure S4: The binding of P10 to PC3 and DU145 cell lines is cell cycle-dependent, essentially occurring only during the G2/M phase.

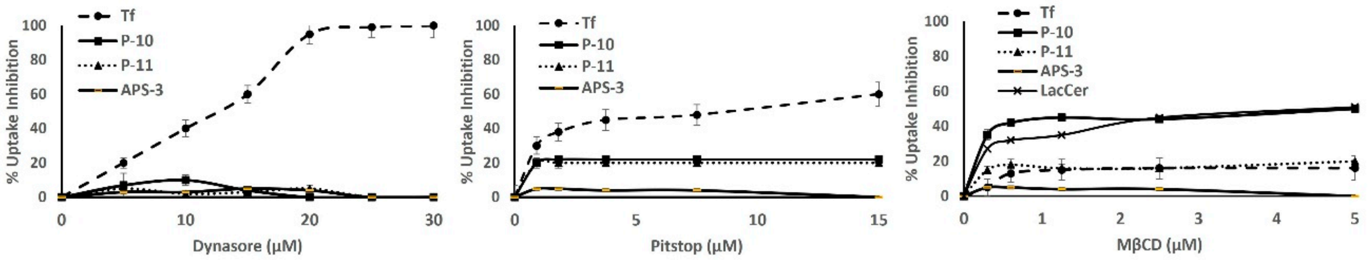

Figure S5: Inhibition of uptake of relevant positive control, P10, P11 or a non-relevant peptide (APS-3) in the presence of increasing concentrations of chemical inhibitors of endocytic pathways Dynasore, Pitstop-2 or MβCB. The data points represent the mean +/- standard deviation of at least 3 repeat experiments, each one run in triplicate.

<u>Optimal conditions of siRNA knockdown of endocytotic proteins</u>

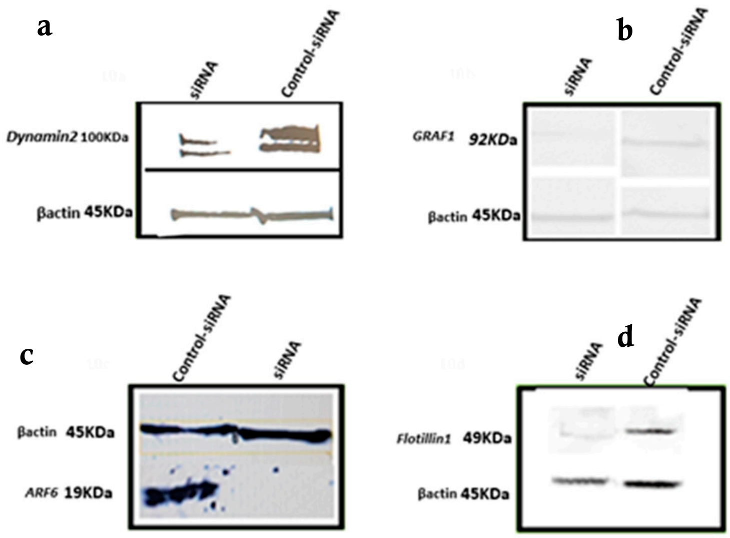

e

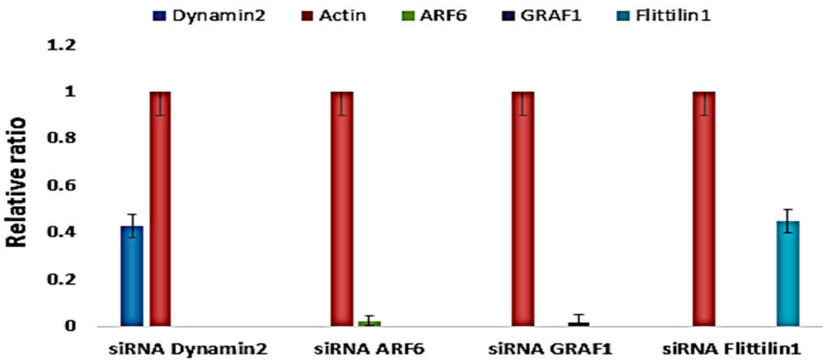

Figure S6: Immunoblotting: (**a-d**) Immunoblots of the optimal conditions for knocking down expressions; (**e**) analysis and relative ratios of specific siRNA/control siRNA treatment cells.

Synthesis of Peptide- Drug-Conjugates

1. Synthesis of P10-Camptothecin

Prodrug synthesis

To prepare the drugs for conjugation, CPT was reacted with 4-Nitrophenyl chloroformate (pNPC) (Alfa-Aesar) to obtain the 4-nitrophenyl carbonate derivative of CPT - the final compound with a carbonate site, see [1–3]. CPT was dissolved in Dichloromethane (DCM) (Biolab, Jerusalem) and cooled to 2–4 °C, under Nitrogen conditions, supplemented by 1.5 equivalents DMAP in DCM as a base. Stirred CPT was reacted with pNPC dropwise for 30 min, till the solution became clear. The product was washed with HCl 3 times to neutralize the base and dried with saturated brine (Sodium Sulfate in DCM). DCM was evaporated and activated crystalized drug was washed with Diethyl Ether, −20 °C. Finally, the CPT was filtered and dried, and stored till coupling procedure with the peptide. The CPT derivative, compound **1**, was tested on LC-MS to verify completion of reaction and product purity.

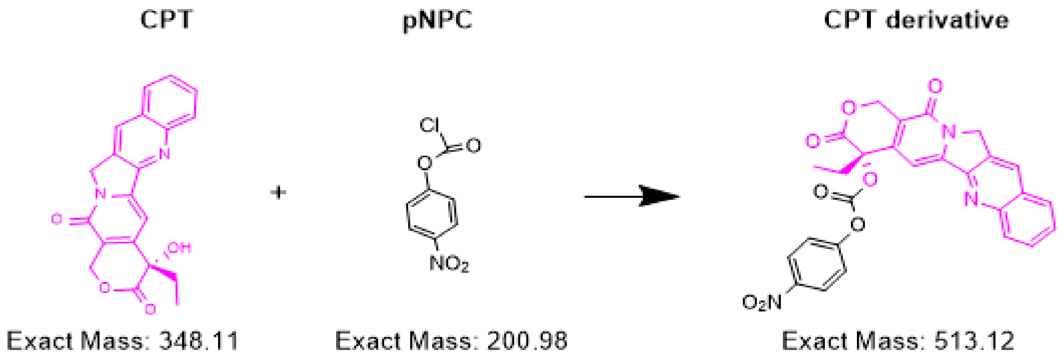

Figure S7: CPT drug reaction with + pNPC resulting in 4-nitrophenyl carbonate derivative of CPT.

Coupling of Peptides

Peptides for the conjugates were synthesized together with Ahx linker on Wang resin from Pepmic (Suzhou, China). Loading 0.93 mmol/g 100–200 Mesh 1%DVB. N-terminus of the Ahx was protected by F-moc. Coupling of the peptide to activated drug began with F-moc removal from the peptide by treatment with 20% Piperidine in DMF by shaking in a reactor (5ml syringe with filter), 2 × 15 min, and subsequent washing with DMF (5 × 2 min) and in DCM (2 × 2min). A solution of activated CPT (1.5 equivalents) in dry DMF and DIPEA (6 equivalents) was added to the reactor and the mixture was shaken in for 1.5 hr. The solvent was removed by filtration and washings in DMF (5 × 2 min) and DCM (5 × 2 min). The resin with conjugate was dried for 30 min by vacuum, transferred to a glass vial. Cleavage of conjugate from the resin was performed with 95% Trifluoroacetic acid (TFA) 2.5% Triisopropylsilane (TIS) 2.5% H_2O cocktail for 1.2 hr. The solution was transferred to a new syringe and filtered through a 0.22um PVDF 13mm filter into an eppendorf vial, evaporated with N_2 and then frozen Diethyl Ether was added to precipitate the conjugate. Following centrifugation for 5 min, 3400× *g*, supernatant was discarded and the sediment dissolved in ACN:H_2O 1:1 and purified by preparative high-performance liquid chromatography (HPLC). The PDC was dried in a lyophilizer for 3 days.

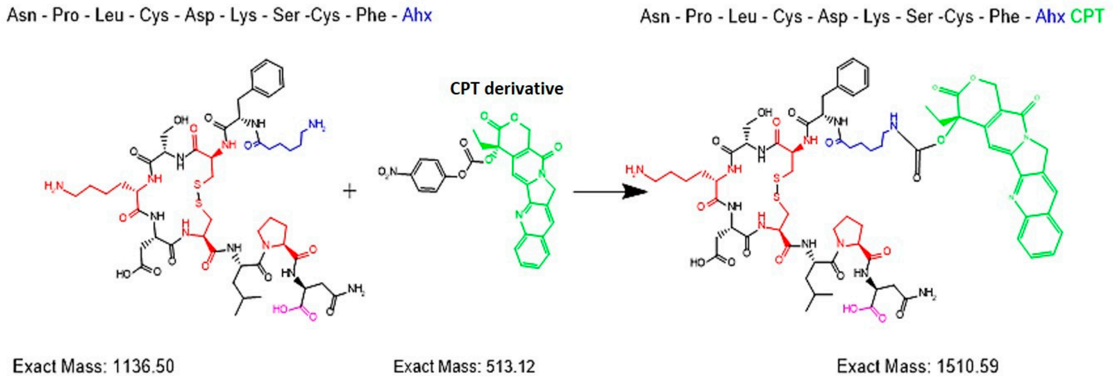

Figure S8. Peptide P10 with Ahx spacer was reacted with a CPT derivative with carbonate ester site, resulting in Peptide P10-CPT PDC.

Compound purity at each step was evaluated on a Dionex Ultimate 3000 chromatograph equipped with VWD (variable wavelength detector) flow cell in line with ISQ quadrupole mass spectrometer (MS, Thermo Fisher Scientific, Austin, TX, USA) The Ultimate 3000 chromatograph was equipped with Diode Array Detector by Thermo (λ = 210 nm) and a Syncronis C18 100 × 2.1 mm, 3 µm column (Bargal), at temperature 50 ºC. The eluent, H_2O and ACN with 0.1% Formic acid was flowed at 0.4 mL/min rate.

<u>Conjugate purity was analyzed by LC-MS</u>.

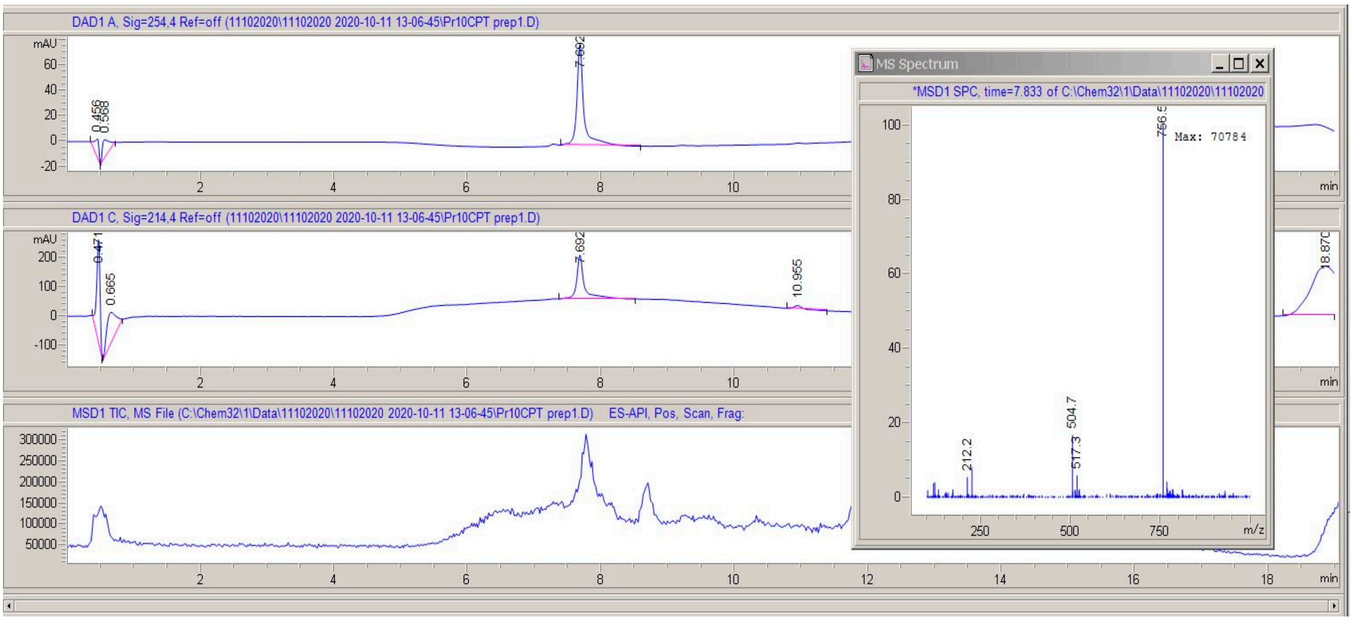

Figure S9a: LC-MS output of the P10CPT conjugation. UV peak at 7.692min at 254nm, MS peak at 756.5 m/z (1510.59/2+1)

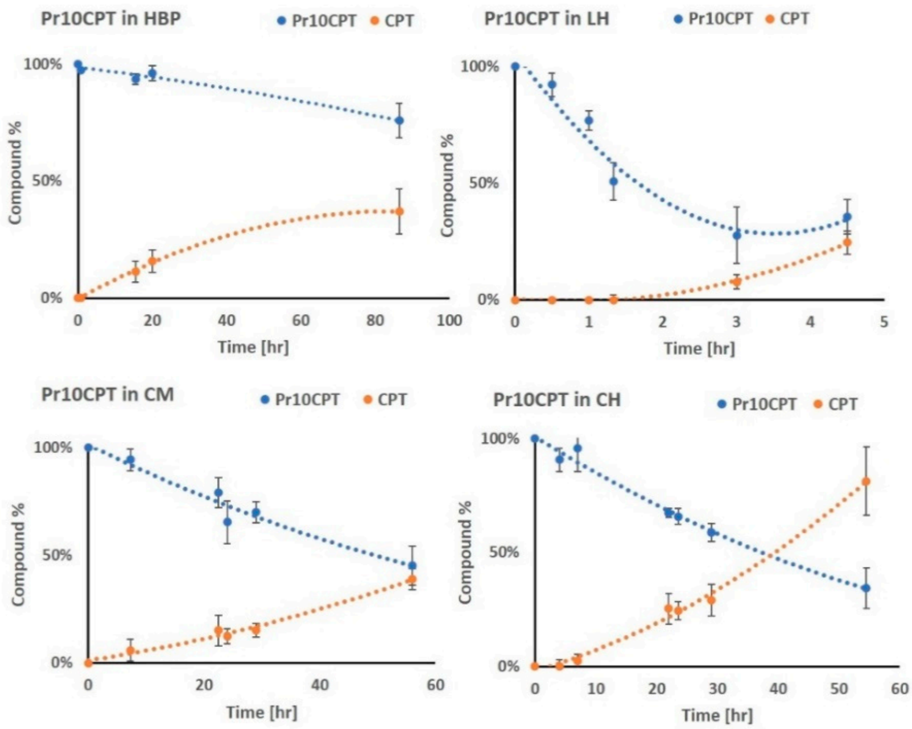

Figure S9b: Chemical and biological stability of the P10-CPT PDC. The biostability of P10-CPT PDC was tested by incubating it in human blood plasma (HBP), mouse liver homogenate (LH) in culture medium (CM) and in PC3 cell homogenate (CH). and monitoring the appearance of free CPT over time.

2. Synthesis of P10-SN-38

Synthesis of Boc-SN38-PNC

The phenolic hydroxyl group of SN38 was protected with Boc group to obtain **Boc-SN38** and subsequently reacted with 4-Nitrophenylchloroformate to get yellow solid **Boc-SN38-PNC** with 75% overall yield (**Scheme** S1), following the procedure outlined in the literature [1].

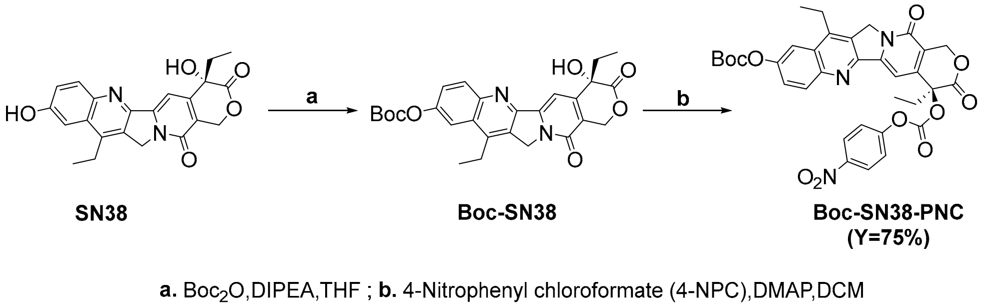

Scheme S1. Synthesis of Boc-SN38-PNC

Solid-Phase Synthesis of Peptide P10

The S-S bridged cyclic peptide P10 was synthesized by solid-phase peptide synthesis (SPPS) on Fmoc-Rink Amide AM resin (RA, loading 0.511 mmol/g, 100–200 mesh, aapptec, USA), as conjugated to SN38 through ε-aminocaproic acid (Ahx) linker using standard Fmoc protocols [2]. The cyclization (**Scheme S2**) between two cysteine amino acids of the peptide was carried out prior to the conjugation step using iodine (10 eq.) in 2% Anisole/ DMF (v/v) for 2 h [3]. The purity of the peptides was no less than 95% (according to HPLC data measured at 254 nm, see Supplementary).

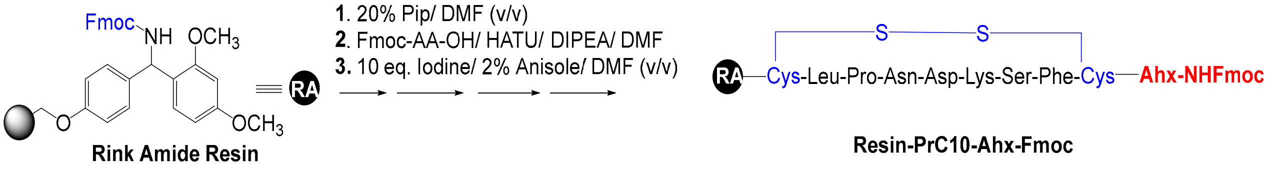

Scheme S2. SPPS of P10-Ahx-Fmoc

Peptide Conjugation to SN38

After getting the Fmoc-Protected cyclic peptide on the resin, the Fmoc protecting group was removed (20% piperidine in DMF, 2 × 10 min), the resin was washed with DMF (3 × 5 min). Then the Boc-SN38-PNC (1.2 eq.), DIPEA (2 eq.) in DMF was dumped onto the resin and Shaked for 8h, washed with DMF (3 × 5 min) and cleaved from the resin by treating with a cold TFA cocktail (95% TFA, 2.5% TIPS, 2.5% H_2O) during the 2 h (**Scheme S3**). Then, the solvent was evaporated by nitrogen and the crude PDC was purified by preparative HPLC on an ECOM preparative system, with dual UV detection. A Phenomenex Gemini® 10 µm RP18 (250 × 21.2 mm) column was used. The column was kept at 25 °C. Eluents A (water) and B (CH_3CN) were used. A typical elution was a gradient from 100% A to 100% B over 42 min at a flow rate of 22 mL/min. **PrC10-Ahx-SN38** conjugate: (85 mg, 22% overall yield, purity 95%); $\mathbf{RT}_{\mathbf{LC/MS}}$ = 5.982 min; **HRMS** for $C72H95N15O20S2^{+}$ calc. 1554.6398; found 1554.6563, for $C72H95N15O20S2^{+2}$ calc. 777.8238; found 777.8260.

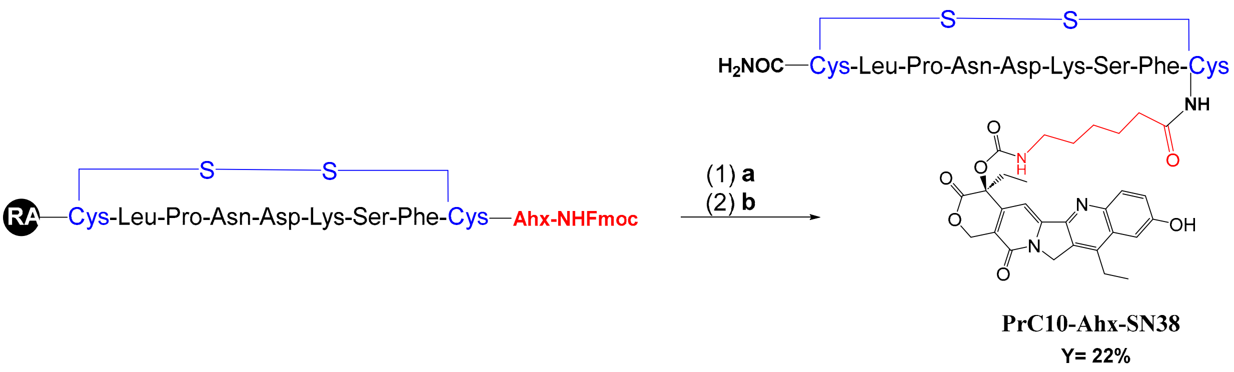

a. (i) 20% Pip/DMF,**(ii)** Boc-SN38-NPC,DIPEA,DMF,8h, rt; **b.**TFA/TIPS/H_2O (95:2.5:2.5),2h,rt

Scheme S3. Synthesis of P10-Ahx-SN38

Material and methods

2.1. General

All chemicals were reagent grade quality from purchased commercial sources without further purification. The amino acids were purchased from Sigma Aldrich. Other amino acids, the coupling reagent 1-[Bis(dimethylamino)methylene]-1*H*-1,2,3-triazolo[4,5-*b*] pyridinium 3-oxide hexafluorophosphate (HATU), and the Fmoc-Rink Amide AM Resin (RA) were purchased from Bachem (Israel). The reagents methanol (MeOH), dichloromethane (DCM), dimethylformamide (DMF), acetonitrile (AcCN), and anhydrous ethyl ether were obtained from BioLab (Israel). Trifluoroacetic acid (TFA), dimethyl sulfoxide (DMSO), triisopropylsilane (TIS), piperidine (Pip), and N, N-diisopropylethylamine (DIPEA) were purchased from Sigma Aldrich (Israel). Yields refer to chromatographically purified compounds. NMR spectra were recorded on a Bruker spectrometer operating at 400 MHz and101 MHz for ^1H and ^{13}C acquisitions, respectively. Chemical reactions were monitored by TLC (Silica gel 60 F-254, Merck) and by LC/MS. LC/MS analyses were performed on an Agilent Technologies 1260 Infinity (LC) 6120 quadruple (MS), column Inertsil ODS-4, 2 µm, 3.0 × 100 mm, column temperature 50 °C, eluent: water — acetonitrile (ACN) contained 0.1 % of formic acid, UV detection at 270, 254 and 214 nm. HPLC purifications were done on an ECOM preparative system, with dual UV detection at 254 nm and 214 nm. Phenomenex Gemini® 10 µm RP18 110 Å, LC 250 × 21.2 mm column was used. The column was kept at 25 ºC. Eluent A (water) and B (CH_3CN) were used. A typical elution was a gradient from 100 % A to 100 % B over 42 min at a flow rate of 22 mL/min. HRMS was performed in ESI positive mode by using an Agilent 6550 iFunnel Q-TOF LCMS instrument.

Solid Phase Peptide Synthesis

<u>Peptide Synthesis</u>

Rink Amide resin (0.255 mmol, 500 mg, 0.511 mmol/g) was swollen in DMF for 2 h in a jacketed fritted vessel. The Fmoc group was deprotected using 20% piperidine/DMF under nitrogen for two times (10 min × 2) and washed with DMF (3 × 5 min). The peptide was assembled on the resin following coupling of amino acid in the order Fmoc-Cys(Trt)-OH (0.51 mmol, 299 mg), Fmoc-Leu-OH (0.51 mmol, 181 mg), Fmoc-Pro-OH (0.51 mmol, 172 mg), Fmoc-Asn(trt)-OH (0.51 mmol, 305 mg), Fmoc-Asp(OtBu)-OH (0.51 mmol, 210 mg), Fmoc-Lys(Boc)-OH (0.51 mmol, 239 mg), Fmoc-Ser(OtBu)-OH (0.51 mmol, 196 mg), Fmoc-Phe-OH (0.51 mmol, 198 mg), Fmoc-Cys(Trt)-OH (0.51 mmol, 299 mg) and Fmoc- εAhx-OH (0.51 mmol, 181 mg) by using HATU (1.8 eq, 0.459 mmol, 175 mg) as coupling agent and subsequent Fmoc deprotection by 20% Piperidine in DMF in all the cases. The coupling reactions were monitored by LCMS.

<u>Cyclization</u>

To the resin, a solution of Iodine (10 eq, 2.55 mmol, 324 mg) in 10 mL 2% Anisole in DMF was added under nitrogen. Then the reaction mixture was shaken by orbital shaker for 2 h, followed by the removal of solvent

by filtration and further washings with DMF (3 × 3 mL) and then DCM (3 × 3 mL). The reaction was monitored by LCMS which shows complete conversion of linear peptide.

SN38 Conjugation

The Fmoc group was deprotected using 20% piperidine/DMF under nitrogen for two times (10 min × 2) and washed with DMF (3 × 5 min). A solution of Boc-SN38-PNC (1.2 eq, 0.306 mmol, 202 mg) and DIPEA (2 eq, 0.1 mL) in 10 ml DMF were added to the resin under nitrogen and Shaked for 8 h under dark. After completion the resin was washed with DMF (3 × 5 min). cleaved from the resin by treating with a cold TFA cocktail (95% TFA, 2.5% TIPS, 2.5% H_2O) during the 2 h.

Supporting information

1. LCMS of PrC10-Ahx-SN38

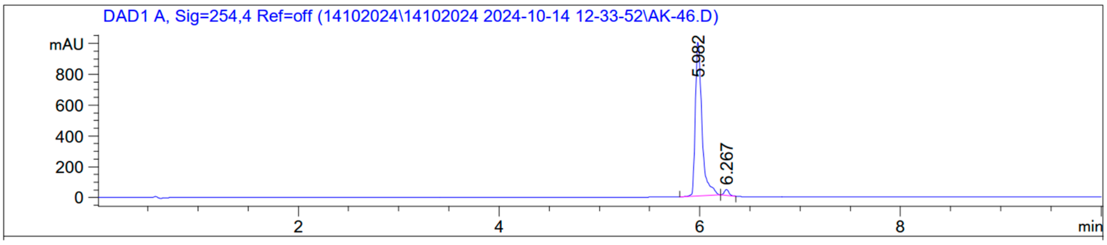

2. HRMS of PrC10-Ahx-SN38

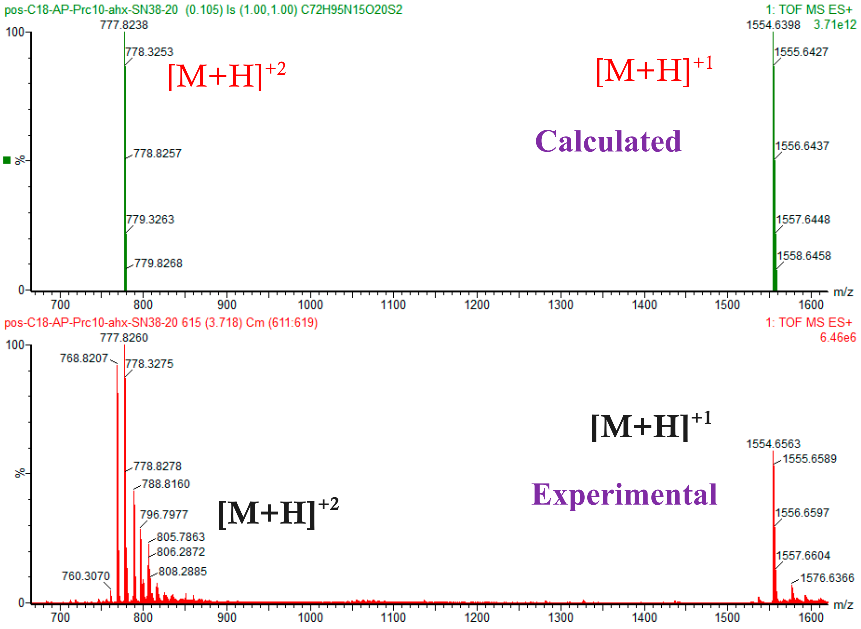

References

(1) He, W.; Du, Y.; Wang, T.; Wang, J.; Cheng, L.; Li, X. Redox Responsive 7-Ethyl-10-Hydroxycamptothecin (SN38) Lysophospholipid Conjugate: Synthesis, Assembly and Anticancer Evaluation. *International Journal of Pharmaceutics* **2021**, *606*, 120856. https://doi.org/10.1016/j.ijpharm.2021.120856.

(2) Furman, O.; Zaporozhets, A.; Tobi, D.; Bazylevich, A.; Firer, M.A.; Patsenker, L.; Gellerman, G.; Lubin, B.C.R. Novel Cyclic Peptides for Targeting EGFR and EGRvIII Mutation for Drug Delivery. *Pharmaceutics* **2022**, *14*, 1505 https://doi.org/10.3390/pharmaceutics14071505

(3) Zhang, H. B.; Chi, Y. S.; Huang, W. L.; Ni, S. J. Total Synthesis of Human Urotension-II by Microwave-Assisted Solid Phase Method. *Chinese Chemical Letters* **2007**, *18* (8), 902–904. https://doi.org/10.1016/j.cclet.2007.06.002.
